# Virtual Reality Therapy for Depression and Mood in Long-Term Care Facilities

**DOI:** 10.3390/geriatrics6020058

**Published:** 2021-06-04

**Authors:** Kevin Zhai, Azwa Dilawar, Mohammad S. Yousef, Sean Holroyd, Haithem El-Hammali, Marwa Abdelmonem

**Affiliations:** 1 Premedical Unit, Weill Cornell Medicine-Qatar, Cornell University, Doha P.O. Box 24144, Qatar; kez4003@qatar-med.cornell.edu (K.Z.); azd4001@qatar-med.cornell.edu (A.D.); msy2001@qatar-med.cornell.edu (M.S.Y.); 2 Department of Physiology and Biophysics, Weill Cornell Medicine-Qatar, Cornell University, Doha P.O. Box 24144, Qatar; smh2010@qatar-med.cornell.edu; 3 VCU School of the Arts in Qatar, Virginia Commonwealth University, Doha P.O. Box 8095, Qatar

**Keywords:** depression, virtual reality (VR), virtual reality therapy (VRT), long-term care facility (LTCF), mood disorder, place attachment, neuro-architecture

## Abstract

Virtual reality (VR) describes a family of technologies which immerse users in sensorily-stimulating virtual environments. Such technologies have increasingly found applications in the treatment of neurological and mental health disorders. Depression, anxiety, and other mood abnormalities are of concern in the growing older population—especially those who reside in long-term care facilities (LTCFs). The transition from the familiar home environment to the foreign LTCF introduces a number of stressors that can precipitate depression. However, recent studies reveal that VR therapy (VRT) can promote positive emotionality and improve cognitive abilities in older people, both at home and in LTCFs. VR thus holds potential in allowing older individuals to gradually adapt to their new environments—thereby mitigating the detrimental effects of place attachment and social exclusion. Nevertheless, while the current psychological literature is promising, the implementation of VR in LTCFs faces many challenges. LTCF residents must gain trust in VR technologies, care providers require training to maximize the positive effects of VRT, and decision makers must evaluate both the opportunities and obstacles in adopting VR. In this review article, we concisely discuss the implications of depression related to place attachment in LTCFs, and explore the potential therapeutic applications of VR.

## 1. Overview

Virtual reality (VR) is the use of various technologies to digitally simulate or recreate an environment in which an observer can realistically hear, see, and/or feel as though they are a part of the simulation [1]. This dynamic perceptualization is achieved through technologies such as wearable head-mounted displays (HMDs), noise cancellation headsets, and other multimodal stimuli to immerse the wearer in the virtual atmosphere [1]. While VR has been a critical training tool in the fields of aviation, military combat, and surgery, it has recently shifted towards clinical psychology and therapy [2]. In fact, with the decreasing costs and increasing accessibility of digital media, VR has significant potential in reconstructing conventional approaches to patient care [3].

VR is a popular topic in psychological research studies for its potential in treating various mental disorders such as schizophrenia, post-traumatic stress disorder (PTSD), anxiety, and depression. Psychotherapy experts rank VR as one of the top psychological interventions with an overall positive growth projection in the coming decades [4]. Rothbaum and colleagues first assessed the clinical effects of VR therapy (VRT) on acrophobic college students [5]. Their study established the efficacy of VRT for trauma and anxiety, and provided a basis for further studies on other psychiatric illnesses [6]. With its ability to continuously expose patients to traumatic experiences or threats on a regular basis, VR can train patients to cope with, and eventually eliminate, the fears and negative emotions associated with stressful experiences [6]. One particular demographic that could benefit from the psychological and therapeutic effects of VR is older individuals who reside in long-term care facilities (LTCFs).

An increasing number of older people prefer to age-in-place until they become unable to care for their needs [7]. The choice of moving to a LTCF, however, often becomes inevitable at a certain stage due to deteriorating health conditions. This inevitability, along with demographic changes due to continuous increases in life expectancy, has become a concern [8]. Scholars such as Schwarz and Brent, argue that the proportion of people aged 80 years or older is expected to triple by 2040 [7]. In the United States of America, the age structure of the overall population is projected to change tremendously in the next decade: from 13 percent of the population aged 65 and older in 2010 to around 20 percent in 2030 [9]. Hence, there is a need for social infrastructure to accommodate the physical and psychological needs of this growing population that may transition into LTCFs.

Transitioning into a LTCF may cause severe distress and amplify pre-existing mental health impairments, corresponding to a higher rate of depression of nursing home residents when compared to non-residents [10]. Furthermore, age-related cognitive decline and desires for a familiar environment can further an older individual’s mental deterioration. As mental illnesses can exacerbate other underlying health conditions such as cardiovascular diseases, afflicted individuals may require more demanding and attentive care [11]. VR can serve as a tool to aid the adjustment of older people into new LTCF environments by immersing them in a familiar space to relieve stress, or by familiarizing them with their new surroundings with continuous exposure. VRT can, therefore, mitigate the negative mental health trends seen in individuals in care homes.

This review article explores the potential of VRT in improving mood and alleviating depression in LTCF residents. We first overview the challenges faced by older individuals transitioning to LTCFs. Place attachment bonds are of particular interest, as the disruption of these bonds is a major cause of depression in care homes. Next, we discuss the clinical features and neurological mechanisms of depression related to place attachment, and highlight the difficulties in diagnosing and treating depression in the LTCF setting. We then concisely review the effects of VRT on mood and depression in older individuals—especially those in LTCFs—and conclude by discussing the benefits and limitations of the implementation of VRT in LTCFs.

## 2. Place Attachment in LTCFs

An individual’s environment shapes their view and perception of life, and therefore plays a critical role in their psychological well-being. Systematic reviews indicate that most older individuals prefer to remain in their current residences rather than transfer into facilities [12]. Alongside the other physical health impairments associated with old age, transitioning from the comfort of their homes into LTCFs causes detrimental cognitive responses in older people. Nevertheless, long-term institutional care is necessary for some older individuals due to the severity of an illness or the lack of an attentive caregiver at home [7]. Moving to a LTCF is extraordinarily stressful and involves nostalgia [13]. In environment–behavior research, nostalgia is considered a result of *place attachment*, an emotional bond that links a person to a place [8]. A common mechanism that explains this bond is proximity-maintaining behavior [14,15]. As previously mentioned, individuals who exhibit strong attachment to certain places are likely to disregard important opportunities just to physically remain in those places [16].

A newly admitted LTCF resident faces a number of challenges associated with forming new social connections, inadequate privacy, adapting to a new institutional setting, and lack of homelike qualities [17]. Attachment to one’s home complicates the transition into LTCFs. The degree of place attachment and the person’s readiness to adapt to a new environment influence the smoothness of the transition and consequent stress levels. Bernard and Rowles argue that relocating to a LTCF can be traumatizing for some people due to their inexperience in changing homes throughout their life [14]. Jones et al. defined a *transition* as a passage between two relatively stable periods of time, in which an individual moves from one life phase to another. A *transition* is a process in which the person must develop new skills, relationships, and coping strategies [14,18].

Manzo and Wright shed light on the relationship between place attachment theory and the ability to transition into a new place. They suggest that in the era of increased mobility, place attachment theory can be used to distinguish mechanisms through which people become attached even to alien places [15]. As Bernard and Rowles wrote, “*theories of place attachment and identity emphasize domains of belonging in a more process-oriented and differentiated way. Place attachment is not only related to attitudes, but also to a gamut of processes operating when people form affective, cognitive, behavioral, and social bonds to the environment, thereby, transforming a space into a place*” [14]. Place attachment dynamics involving interrelated processes between people and places are called *place processes*. Examples of these processes include *place identity*, a process in which people view a place as a significant part of their world, and *place interaction*, which refers to the typical going-ons in a place [15]. In a well-liked place, all place processes are sophisticatedly interconnected; feeling at-home is being *fit* in a certain place and being able to continuously engage in place-making processes [15]. In other words, the degree of place attachment pre-determines a person’s willingness and ability to transition into a new place such as a LTCF.

## 3. Depression in LTCFs

### 3.1. Causation and Neural Mechanisms of Depression in LTCFs

As discussed above, the transition into a LTCF is a significantly stressful event for older people, especially during the first four weeks following admission [19]. A major reason for this transitional stress is the disruption of place attachment bonds that play a major role in an individual’s psychological development and well-being. According to Scannell and Gifford, maintaining proximity to one’s place of attachment can be beneficial in many respects, as this provides emotional healing, cognitive restoration, belongingness, and escape opportunities from daily stressors [20]. Relocation to an entirely foreign place, without the trappings of home and family, can be psychologically devastating. As such, Texas senior home residents reported losses of independence, disruptions of continuity with their past (familiar) lives, and loneliness to be precipitating factors (stressors) in their negative moods and/or depression [10].

The *diathesis–stress model* of mental disorders holds that certain individuals have genetic, anatomical, and/or psychological predispositions that remain dormant until activated by a source of stress [21]. Predispositions for depression include alterations in brain structure, especially in the bilateral amygdala, hippocampus, and dorsolateral prefrontal cortex (PFC). These neuroanatomical changes have been found in first-degree relatives of patients with depression, suggesting a mechanism of risk [22]. Moreover, stressful events, such as serious housing problems and separation from friends or loved ones, are causally related to depression [23].

**Figure 1:**
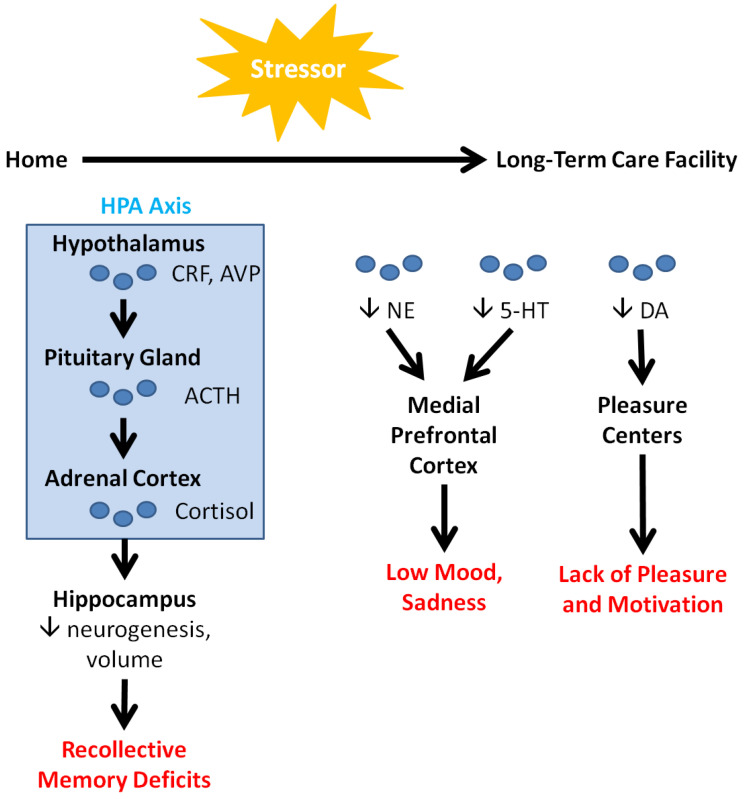
Neurological factors in depression. Acute and chronic stressors, such as disruptions in place attachment bonds, activate the HPA axis and modulate neurotransmitter levels, leading to cognitive and emotional consequences (highlighted in red). These consequences are alleviated in older people by VRT (see Table 1), encouraging the use of VRT with LTCF residents.

Depression is characterized by symptoms such as persistent disturbances of mood, loss of interest in activities, sleep abnormalities, feelings of guilt, loss of energy, impaired concentration, changes in appetite, psychomotor agitation, and in some cases, suicidal ideation [22]. These psychological and cognitive symptoms may manifest as a result of neurochemical and neuroanatomical changes precipitated by stress. As illustrated in Figure 1, transitional stress immediately activates the hypothalamic–pituitary–adrenal (HPA) axis. The hypothalamus releases corticotropin release factor (CRF) and vasopressin (AVP), which stimulate the release of adrenocorticotropic hormone (ACTH) from the pituitary gland. ACTH causes the adrenal cortex to release cortisol, the *stress hormone* [24,25]. Among other functions, cortisol downregulates hippocampal neurogenesis, leading to a decrease in hippocampal volume and consequent memory deficits [25].

Over a longer term, stressors in LTCFs decrease the levels of circulating neurotransmitters (NTs) such as norepinephrine (NE), serotonin (5-HT), and dopamine (DA). NE and 5-HT ordinarily interact with the medial PFC to regulate mood, sleep, appetite, and other bodily functions. Consequently, downregulation of these NTs leads to the low mood and sadness that are characteristic of depression [26]. These NTs also interact with the amygdala, the portion of the brain responsible for emotions and fear conditioning. Their dysfunction can lead to anxiety, which often co-occurs with depression. In contrast, DA is heavily implicated in learning and the reward system, acting in conjunction with the brain’s pleasure centers. DA’s downregulation correspondingly decreases pleasure and intrinsic motivation, leading to a general loss of interest [26]. Finally, a clinical study on patients with late-life depression by Smith and colleagues detected increased levels of *N*-acetylaspartate in the posterior cingulate cortex. They also found associations between decreasing levels of glutamate and glutathione, and decreasing symptom severity [27].

While the neural mechanisms of depression are well established, measuring depression in LTCF residents can be a difficult task. As stress is a major causal factor in depression in LTCF residents, measurement of the levels of cortisol, the hormone associated with stress, would ideally be able to confirm diagnosis. Blood cortisol levels can be matched to the degree of depression in some cases of acute and severe depression [28,29]. However, in mild, chronic, or atypical depression, variability exists in the responsiveness of the HPA axis to stressors [30,31,32,33]. As such, other factors may be at play with regards to the HPA axis’ relationship with depression; these include changes in the glucocorticoid receptors, the mineralocorticoid receptors, or the responsiveness of the axis to AVP and CRF [33]. Therefore, the hormone cortisol cannot be used alone as a marker of depression. Studies have suggested that the levels of various NTs and other biomarkers could support the diagnosis and categorization of depression [34,35]. While these theories are promising, further research is needed to make biomarker identification a viable option to diagnose depression [35].

The lack of a simple laboratory test to aid the diagnosis of depression means that other methods are necessary [36]. While many depression-identifying tests have been validated, they have varying sensitivity [37,38,39]. These tests include questions related to physiological variables such as eating habits, weight loss, and sleep, as well as psychological variables such as mood, behavior, and thoughts. Tests can range from short self-reporting tests to detailed interviews involving highly trained medical staff [40,41]. Self-reporting tests are advantageous as they can be administered *en masse*; as such, they are time-friendly and provide extended coverage, but their accuracy is debatable [42]. There is general agreement in the literature that one-on-one interviews with clients by trained professionals are more exact in diagnosing depression. This accuracy, however, is fully dependent upon the training of those administering the test as well as the amount of time required for each test. These constraints, alongside a lack of administrative support, limit this option in LTCFs [38].

Currently accepted numbers of those with depression in LTCFs are based on a mixture of results from varied tests run by different researchers or organizations on wide-ranging populations. Without a specific biomarker accepted for the diagnosis of depression, multiple depression-identifying tools—including surveys—are heavily relied upon. Self-reporting tests may be used to identify LTCF residents suspected of suffering from depression. However, it is recommended that individuals are then evaluated by trained professionals [36]. Obtaining a true representation of depression in LTCFs requires coordinated mechanisms for standardized testing throughout the whole LTCF community.

### 3.2. Implications of Depression in LTCFs

Critically, depressive symptoms are present in one third of the older population, with the proportion of depressed older people residing in nursing homes being considerably higher than their community-dwelling counterparts [10,43]. As such, depression and anxiety are the most common mental disorders among older adults in LTCFs, yet have been inadequately studied in social research [44]. Notably, depression in LTCFs is customarily linked to the aging process and therefore overlooked. Important mental effects of depression include reduced well-being, loneliness, functional impairment, and increased mortality [44]. Moreover, depression is a major contributing factor to suicide among older people. In fact, suicide rates among older people are generally higher than those for younger people worldwide [45]. The latter fact is alarming and underscores a need for environment–behavior research concerning the environmental causes of depression in LTCFs.

A major barrier to depression research and treatment in LTCFs are the physical and psychological comorbidities of depression in older people. Age-related depression occurs along with other illnesses, such as dementia, chronic conditions, and functional limitations [46]. The management of depression in people with dementia is particularly challenging as neuronal degeneration complicates treatment responses and prolongs hospitalization [47]. Recovery and rehabilitation are further complicated by depression’s direct relationship with physical illness [48]. Depression may affect cardiovascular endurance: Guinjoan et al. concluded that autonomic function is altered in major depression, indicating decreased parasympathetic activity and increased sympathetic activity. These alterations have implications for the increased risk of cardiac disease found in patients with major depression [49]. In a recent study, depression was found to be associated with decreased heart rate variability (HRV), which is a predictor of cardiovascular morbidity [50].

The co-occurrence of depression with dementia in older individuals poses significant challenges with regards to treatment. Antidepressants constitute a canonical therapeutic modality; however, they alter the structure and function of various brain regions, leading to detrimental neural and cognitive side effects that are particularly problematic in those with dementia [47]. Psychosocial treatments such as reminiscence and behavior therapy are constrained by their limited efficacy [47]. As such, it is necessary to explore technologically-driven treatment modalities, such as VRT, in alleviating depression and place attachment in LTCF residents.

## 4. VR’s Effect on Depression and Mood in Older People

The medical applications of VR have been extensively reviewed [51]. Applications span numerous disciplines—including medical education and training, robotic surgery, molecular biology, forensic pathology, and others [52,53]. We will focus primarily on the applications of VR in psychology, specifically as a therapeutic intervention for depression and mood in LTCFs. Given the limitations of behavioral therapy due to the complex psychological states of older individuals, the application of VR for mental health treatment is critical.

It is evident that depression is associated with increased stress levels related to place attachment and relocation. A number of studies investigated reminiscence therapy using digital media and new technologies, such as VR, as a treatment for memory loss in people with dementia [54]. In the cognitive and behavioral sciences, VR is defined as “*an advanced form of human–computer interface that allows the user to interact with and become immersed in a computer-generated environment in a naturalistic fashion*,” meaning that patients can “live” the experience of being in completely different spaces during the VR session (telepresence) [55]. However, the use and implementation of the technology is debated amongst researchers and healthcare practitioners.

Describing the spectrum and limitations of VR technologies, Klein et al. write that “virtual reality worlds with a high level of interaction demands are often too hard to grasp for people with dementia in terms of cognitive elaboration and comprehension of the metaphors used. Nevertheless, there is indication that simulated locations and objects can have a positive impact for reminiscence therapy” [54]. Such positive effects of VR are demonstrated by a number of recent cognitive and psychological studies. For instance, Yang and colleagues examined the impact of VR technology on falls and depression among older individuals with mild depression, with their results showing that depression and internal stress scores were reduced after the intervention. They concluded that the VR exercise program exerted a positive effect on the psychological function of the aged and could be potentially utilized as a therapeutic intervention for reducing depression and internal stress among older people [56].

While literature of VRT as an intervention for depression are currently limited, numerous recent trials demonstrate the value of VRT in enhancing moods and cognition in older people (Table 1). These include several ambulatory studies. For instance, Chan et al. studied 236 healthy members (aged 60 and above) of community centers in Hong Kong. Members of the trial group were exposed to a VR-based tour of Hong Kong’s landmark sites, which included both present-day and 20-year-old images. After a single exposure, they exhibited increases in overall positive emotions—including increased interest, enthusiasm, and excitement. They also exhibited decreases in overall negative affect and its components, such as distress, hostility, and guilt [57]. In another study, Graf et al. exposed 14 home-residing pensioners to a VR “forest walk” experience via the Oculus Go headset system. The experience included a virtual dog as a companion, and several cognitively stimulating mini-games. After the VR exercise, participants demonstrated an increased overall positive affect [58]. Moreover, Banos and colleagues exposed 18 Spanish Senior University participants (aged 58 to 79 years) to VR nature walks meant to elicit joy and/or relaxation. After one, two, or three exposures, both the joy-inducing and relaxation-inducing virtual environments (VE) increased perceived joy and relaxation, and reduced sadness and anxiety [59]. These trials underscore the ability of a single VRT session to influence mood among older individuals.

Other longer-term ambulatory studies also corroborate the potential of VRT to induce positive moods among older people. Barsasella et al. exposed 29 Taipei Medical University Aging Center patients (ages 60–94) to VR biweekly for six weeks (twelve total exposures). VR experiences were delivered through several applications on the High-Tech Computer Corporation’s Vive platform. Notably, participants exhibited statistically significant increases in happiness, as measured through the Chinese Happiness Inventory, post-intervention [60]. Additionally, Gamito and colleagues exposed Portuguese senior daycare center users to biweekly VR interventions over the course of six weeks. The intervention was a city-based VR in which participants carried out everyday activities, such as household organization, shopping, and watching television. Intervention group members exhibited increases in general and visual memory as well as attention [61].

Similar results to those of the aforementioned trials were obtained from studies in LTCFs. Brimelow et al. exposed 13 Australian residential aged care facility (RACF) residents, aged 66–93 years, to relaxing scenes through the Samsung Galaxy S7 smartphone-Samsung Gear VR headset system. After a single exposure, participants exhibited decreased total apathy and increased levels of facial expression, eye contact, physical engagement, verbal tone, and verbal expression [62]. In another study by Saredakis and colleagues, 17 RACF residents were exposed twice to a wandering experience based on Google Street View. This VR intervention utilized the Wander application through YouTube VR and the Oculus Go headset. The investigators found correlations between decreased apathy after the VR experiences and increased semantic fluency [63]. Finally, D’Cunha et al. exposed 11 RACF residents to an immersive bicycling experience through projected footage and stationary pedal exercisers. While no significant changes in mood or apathy were observed, the majority of participants enjoyed the experience, which allowed them to reminisce on cycling memories [64].

## 5. Concluding Remarks

Numerous studies substantiate the claim that VRT has clear potential in alleviating negative feelings while promoting improved cognitive ability and positive emotions in older patients. They indicate that VRT reduces overall negative feelings—such as apathy, distress, and anxiety—while increasing overall positive outlook compared to pre-exposed VRT patients [57,58,60]. It is worth noting here that, while anatomical and molecular factors such as NTs were not assessed in the reviewed studies, the described psychological findings suggest that VRT modulates the neurological mechanisms underlying mood and depression (Figure 1). Moreover, not only does VRT correlate with psychological relief, but evidence also suggests that VRT can improve both physical and cognitive functionality in older patients. As shown by Brimelow et al. and Saredakis et al., patients who underwent VRT exhibited improvements in visual memory, linguistic fluency and expression, and physical engagement [62,63]. These heightened abilities can alleviate the feelings of social exclusion felt by many LTCF residents, furthering the positive effects of VR on a patient’s overall psychological well-being. In this light, VRT has the potential to improve mood and cognition, and mitigate the effects of depression—including those related to place attachment—in residents of care homes.

**Figure 2:**
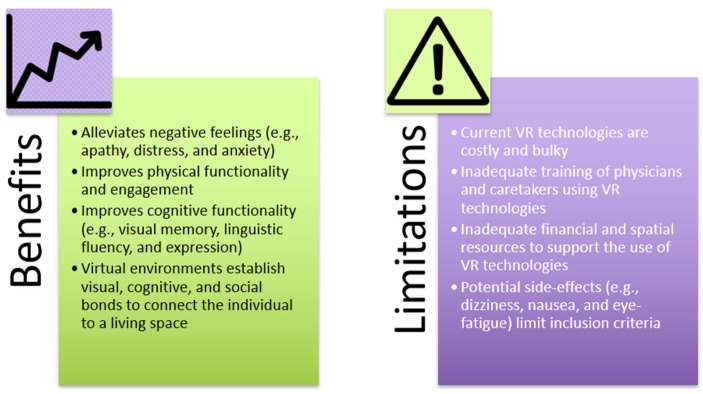
Potential benefits and limitations of the use of VRT in LTCFs.

While VRT offers many benefits, there are various challenges involved in its large-scale implementation in mental rehabilitation and therapy (Figure 2). Current technologies are costly and bulky, limiting their applicability in care homes due to inadequate financial resources or physical space. Furthermore, adequate training for using the technologies is required for physicians and caretakers to maximize benefit from the treatments. VRT has been attributed to various side-effects, including dizziness, nausea, and eye-fatigue, effectively limiting the population who can participate in the treatments [65,66]. Given that older people already have decreased cognitive ability due to mental deterioration, VRT may cause further aggravation and discomfort. Moreover, lack of technological understanding and insufficient physical ability may also make older individuals apprehensive of VRT, making it harder to implement as a therapeutic intervention [67]. In addition to user concern, further research is necessary to consolidate the benefits of VRT so to make it a more accepted long-term intervention within the medical field [68]. In the context of broader future applications of VRT, as well as being a potential treatment for psychological ailments in older people, VRT can also be suggested for other age groups—including children, adolescents, and middle-aged individuals. A recent study by Mesa-Gresa and colleagues yielded positive results for the use of VR for autism and phobias in children and adolescents, exemplifying the extent to which VRT can aid in psychological rehabilitation in various populations [69]. This study found that VRT increased the emotional understanding and enhanced the communication skills of individuals on the autism spectrum. With this, prospects for the future application of VRT for psychological disorders are endless.

Numerous studies support that VR provides an opportunity for LTCF residents to adapt to their new environments in a controlled atmosphere, ultimately alleviating distress and subsequent psychological turmoil, and instead promoting positive feelings and emotions. Virtual environments can elicit positive psychological responses due to their ability to establish the necessary visual, cognitive, and social bonds to connect the individual to the living space. The neuroscientific influences of these virtual environments that simulate real structural environments are profound. The National Human Activity Pattern Survey finds that approximately 90% of an individual’s life is spent within a built system [70]. Built systems are necessary for one’s social, mental, and physical development—ultimately influencing one’s perception and psychological state. As the process of aging starts to deteriorate the cognitive mind, the ability to emotionally connect to a new environment is also hindered, contributing to social exclusion and depression in older individuals. Hence, social planners, architects, neuroscientists, and psychologists must collaborate when designing infrastructures catered to older people due to the influence of these structured environments on psychological well-being [71]. To maximize the positive effects of VRT, proper infrastructure for VR equipment and adequate training of healthcare providers are necessary. Educational programs to destigmatize VR may also prove beneficial for establishing end user trust [67]. Finally, further quantitative and qualitative research is needed in the fields of environmental psychology and environmental neuroscience to explore and substantiate the potential of VR technology in emotional and mental health.

## Figures and Tables

**Table 1:** Effects of VR on mood and cognitive function in older people.

Participants	VR Experience	Study Design	Findings	Source
RACF residents: 13 total (9 females and 4 males); 66–93 years old.	Smartphone-based VR experience with relaxing videos (e.g., nature).	All participants exposed once to the VR experience. Measurements taken before and after.	↓ total apathy ↑ facial expression, eye contact, physical engagement, verbal tone, verbal expression.	Brimelow et al., 2020 [62]
Members of 19 community centers: 236 total (180 females and 56 males); 60+ years old.	VR cognitive stimulation experience: virtual tour of Hong Kong’s famous sites.	Trial group exposed once to the VR experience. Control group exposed to paper-and-pencil cognitive stimulation activity.	↑ total positive affect (e.g., interested, excited, strong, alert, determined) ↓ total negative affect (e.g., distressed, upset, guilty, hostile).	Chan et al., 2020 [57]
RACF residents: 10 total (8 females and 2 males); 75–94 years old.	VR immersive biking experience: included both a video and a stationary pedal system to follow along.	Trial group exposed once to the VR experience. Control group exposed to a standard occupational therapy activity.	No significant measured changes in mood or apathy. Subjective experience: most participants enjoyed the scenery and reminisced about previous biking memories.	D’Cunha et al., 2020 [64]
University Aging Center patients: 60 total (46 females and 14 males); 60–94 years old.	Headset-based VR experience with nine apps, each involving high, low, or very low intensity movement.	Trial group exposed to biweekly VR experiences for 6 weeks. Control group received no intervention.	↑ happiness Greater EQ-5D improvement after VR therapy than in controls	Barsasella et al., 2020 [60]
Daycare center users: 43 total (34 females and 9 males); 67–87 years old	VR cognitive stimulation experience: increasingly complex attention, memory, and executive tasks in a virtual city environment.	Trial group exposed to biweekly VR experiences for 6 weeks. Control group exposed to weekly paper-and-pencil cognitive stimulation for 6 weeks.	↑ general, visual memory ↑ attention	Gamito et al., 2020 [61]
Senior University participants: 18 total (14 females and 4 males); 58–79 years old	Two natural virtual environment (VE) experiences: (1) joy-inducing VE; (2) relaxation-inducing VE.	All participants exposed to one or both VEs; one to three total exposures. Measurements taken before and after.	For both VEs: ↑ joy, relaxation ↓ sadness, anxiety	Banos et al., 2012 [59]
Pensioners residing at home: 14 total (8 females and 6 males); 66–84 years old	Headset-based VR forest walk experience with embedded mini-games.	All participants exposed once to the VR experience. Measurements taken before and after.	↑ total positive affect	Graf et al., 2020 [58]
RACF residents: 17 total (10 females and 7 males); 72–95 years old	Headset-based VR wandering experience based on Google Street View.	All participants exposed twice to the VR experience. Measurements taken before and after.	Correlation between ↓ apathy (after VR exposure) and ↑ semantic fluency	Saredakis et al., 2020 [63]

## Data Availability

Not applicable.
